# In vivo persistence of adoptively transferred TCR gene-transduced lymphocytes with anti-tumor reactivity in patients with MAGE-A4 expressing esophageal cancer

**DOI:** 10.1186/2051-1426-1-S1-O3

**Published:** 2013-11-07

**Authors:** Hiroaki Ikeda, Shinichi Kageyama, Naoko Imai, Yoshihiro Miyahara, Mikiya Ishihara, Naoyuki Katayama, Hirofumi Yoshioka, Daisuke Tomura, Ikuei Nukaya, Junichi Mineno, Kazuto Takesako, Hiroshi Shiku

**Affiliations:** 1 Department of Immuno-Gene Therapy, Mie University Graduate School of Medicine, Mie, Tsu, Japan; 2 Department of Hematology and Oncology, Mie University Graduate School of Medicine, Mie, Tsu, Japan; 3 Center for Cell and Gene Therapy, Takara Bio Inc., Shiga, Japan

## 

The application of adoptive immunotherapy with tumor-specific T cells has been limited because of the short life span of the transferred T cells unless the host has been manipulated. Engineering the antigen receptor gene in patients’ lymphocytes is one promising strategy to create antigen-specific lymphocytes without senescent phenotypes. The strategy provides an opportunity to broaden the types of cancer to be treated. However, this concept has not been tested in the epithelial cancer patients. We completed a phase I clinical trial of TCR gene therapy targeting MAGE-A4 to treat esophageal cancer patients without lympho-depleting pre-conditioning. The trial was designed as a cell-dose escalation consisting of three cohorts, 2x10E8, 1x10E9 and 5x10E9 cells/patient. The treatment was tolerable with no adverse events associated with transferred cells. In all ten patients of the 3 cell-doses, the transferred lymphocytes were detected in their peripheral blood in a dose-dependent manner during the first 14 days. In 4 patients, the infused cells have been persisting more than 5 months after the transfer. The T cell clones were established from the transferred lymphocytes that were harvested more than 100 days after the transfer. These clones sustained the reactivity to the antigen-expressing tumor cells. Three patients showed SD or long tumor free status. These results suggest that this approach may extend the availability of adoptive T cell therapy for epithelial cancer patients by providing tumor-reactive and long surviving lymphocytes reducing the risk of intensive pre-treatments.

